# Impact of Methicillin Resistant *Staphylococcus aureus* Contact Isolation Units on Medical Care

**DOI:** 10.1371/journal.pone.0057057

**Published:** 2013-02-25

**Authors:** Vincent Masse, Louis Valiquette, Soraya Boukhoudmi, Francis Bonenfant, Yasmine Talab, Jean-Christophe Carvalho, Isabelle Alarie, Nathalie Carrier, Paul Farand

**Affiliations:** 1 Infectious Diseases Division, Centre Hospitalier Universitaire de Sherbrooke, Sherbrooke, Quebec, Canada; 2 Internal Medicine Division, Centre Hospitalier Universitaire de Sherbrooke, Sherbrooke, Quebec, Canada; 3 Etienne-LeBel Research Center, Centre Hospitalier Universitaire de Sherbrooke, Sherbrooke, Quebec, Canada; University of Maryland, United States of America

## Abstract

**Background:**

Patient isolation using contact precautions has gained widespread use to halt MRSA transmission, however supportive data is scarce and concerns regarding patient safety and satisfaction have been raised. At our institution, MRSA patients are isolated on a dedicated ward (cohort isolation), rather than in separate rooms. Our objectives were (1) to determine the proportion of bedside medical visits to patients on an isolation ward, (2) to quantify complications in those patients and (3) to determine if those complications are related to isolation and if they can be prevented.

**Methods:**

This retrospective case-control study was performed on the two sites of a tertiary teaching hospital in Sherbrooke, QC, Canada. We matched MRSA patients with an admission diagnosis of heart failure or chronic obstructive pulmonary disease to similar non-isolated controls. The proportion of bedside visits was ascertained through the number of progress notes with subjective elements or with a physical examination. Complications were sought through an extensive file review, and events were analysed according to Baker’s CAES causality and preventability scales.

**Results:**

Overall, 111 patient pairs were analysed (35 with heart failure and 76 with COPD). Isolated patients received less bedside visits (subjective notes/1,000 patient-days: 849.6 *vs.* 983.3, p = 0,001). Attending physicians (454.5 *vs.* 451.4, p = 0,02) and residents (347.0 *vs.* 416.9, p = 0.01) are responsible for this discrepancy, while medical students appear to visit isolated and non-isolated patients equally (116.5 *vs.* 114.9, p = 0.90). Isolated patients showed a tendency towards longer stay and more preventable complications, although no difference in the total number of complications was observed.

**Conclusion:**

Isolated patients have less documented care that suggests less bedside visits from the medical staff, which could hamper the therapeutical relationship. Further studies are needed to explain this finding.

## Introduction

Patient isolation using contact precautions has gained widespread use as a way to reduce propagation of various infectious agents, such as Methicillin resistant *Staphylococcus aureus* (MRSA). Authorities have published specific guidelines on the topic, advocating for active isolation of MRSA patients [1].

Nonetheless, systematic reviews have failed to provide strong support to official recommendations, mostly due to data heterogenicity [2]. Polarised expert opinions have also been published [3], [4]. Moreover, adverse outcomes associated with contact precautions were rigorously highlighted in a 2009 systematic review [5]. However, most, if not all, data available on contact isolation pertain to patients isolated on an individual basis rather than on a special isolation unit.

At our tertiary care centre in Sherbrooke (Canada), patients are screened for MRSA colonisation upon admission, and positive results lead to isolation on a dedicated ward where strict isolation measures are enforced. The rationale behind such cohort isolation (a common practice in our province) is to aggressively limit spread, to reinforce adherence to contact precaution measures, and to make those measures less cumbersome for everyone. However, data on this specific type of isolation are lacking and those alleged benefits do not seem to have been evaluated.

Our objectives were: (1) To determine the proportion of bedside medical visits to isolated patients, (2) to quantify complications in those patients and (3) to determine if those complications are related to isolation and if they can be prevented.

## Methods

### Ethics Statement

The project received approbation from the Ethics committee of health research in humans of the Centre Hospitalier Universitaire de Sherbrooke (CHUS). Considering the retrospective design of this study, and with approval of our institutional review board, we did not obtained informed consent from participants involved.

We conducted a retrospective case-control study using 2 admission diagnoses (heart failure and chronic obstructive pulmonary disease (COPD)), at the Centre Hospitalier Universitaire de Sherbrooke (CHUS) in the province of Quebec, Canada. Our 727-bed, tertiary care teaching hospital located on two distinct sites (Hôpital Fleurimont and Hôpital Hôtel-Dieu).

Using CIRESSS (*Centre Informatisé de Recherche Évaluative en Services et Soins de Santé*), our local data warehouse combining extensive data from the computerized patients’ records with data on diagnoses (ICD-9-CM) [6], we selected patients with one of the two pre-specified admission conditions who were admitted directly to the “MRSA ward” between January 2005 and December 2008 because of known prior MRSA carriage. Charlson comorbidity scores were used to quantify the overall burden of comorbidities [7].

These MSRA isolation units (one per site) are physically separated from the other units by a gate and changing area where clear instructions on hand hygiene and isolation are posted. Isolated patients are free to circulate on this ward, but visitors and personnel are expected to put on a gown once per visit in the unit and to change gloves between patients. Patients themselves must suit-up if leaving the unit for procedures or tests. A total of 32 beds are available for both sites, with a usual nurse: patient ratio of 1∶6. Staff is assigned to the MRSA-unit on a shift-to shift basis, but does not provide care simultaneously to MRSA- negative patients.

In the study. each MSRA patient was matched to a control, non MRSA patient (non isolated on the regular ward) according to admission diagnosis, hospital site, sex, age (+/−5 years), and Charlson comorbidity score (+/−1). Admission dates had to be less than two years apart. A manual chart review was then undertaken by a medical student (YT).

To estimate the number of bedside medical visits (where physicians or trainees physically went to the bedside), we counted the number of medical progress notes that included subjective elements (*i.e*. elements of a discussion or a progress description) and/or physical examination (excluding notes reporting only vital signs, as this information is readily available through the computerised patient chart system without visiting the bedside). We considered these elements as a reasonable retrospective marker of a medical bedside visit. The result was expressed as a number of subjective notes/1,000 patient-days. Multiple daily notes and death reports were excluded from the count. Rates for each group were compared using a Chi-square test. Comparisons were made on raw data (*i.e.* in patient-days, not 1,000 patient-days).

Complications (*i.e.* any new medical situation arising during the hospital stay) were sought after by an exhaustive review of medical and nursing progress notes, blood work, imaging studies and dedicated accident report forms. Total numbers of complications in each cohort were compared using a McNemar test. Every individual complication was then analysed by two independent, blinded senior internal medicine residents (VM, FB) following Baker’s Canadian Adverse Event Study (CAES) grid for causality and preventability [8]. McNemar comparisons were then performed between each group.

## Results

Initially, 120 MRSA patient pairs were found between January 2005 and December 2008. Nine of those could not be adequately matched and were hence excluded, leaving 111 patient pairs for analysis (35 with heart failure and 76 with COPD).

Both groups were similar (Table 1), although controls showed a tendency towards shorter hospital stays (6.0 *vs.* 7.0 days, p = 0.08). The median number of complications was similar (0.57 *vs.* 0.59, p = 0.89).

**Table 1:** Patients Characteristics.

Patients Characterictics	Isolated patients (n = 111)	Controls (n = 111)	p-Value
**Median age, years (IQR)**	80.0 (69.0–86.0)	80.0 (68.0–86.0)	0.92
**Female sex (%)**	44 (39.6%)	44 (39.6%)	1.00
**Admission diagnosis**			
** Heart failure (%)**	35 (31.5%)	35 (31.5%)	1.00
** COPD (%)**	65 (68.5%)	65 (68.5%)	1.00
**Median hospitalisation duration, days (IQR)**	7.0 (3.0–13.0)	6.0 (4.0–10.0)	0.08
**Median Charlson score (IQR)**	7.0 (5.0–9.0)	7.0 (4.0–9.0)	0.37
**Median creatinine clearance, mL/min/1.73 m^2^ (IQR)**	51.9 (35.1–72.4)	51.6 (36.5–74.0)	0.44
**Median left ventricular ejection fraction, % (IQR)**	55.0 (37.5–60.0)	50.0 (30.0–55.0)	0.75
**Mean number of complications per patient, n ± SD**	0.57±1.01	0.59±1.05	0.89

COPD : Chronic Pulmonary Obstructive Disease.

Table 2 shows that isolated patients received fewer medical visits as evidenced by less subjective notes/1,000 patient-days (849.6 *vs.* 983.3, p = 0,001). Attending physicians (454.5 *vs.* 451.4, p = 0,02) and residents (347.0 *vs*. 416.9, p = 0.01) are responsible for this discrepancy, while medical students appear to visit isolated and non-isolated patients equally (116.5 *vs*. 114.9, p = 0.90).

**Table 2:** Assessment of Bedside Visits.

	Isolated patients(n = 111)	Controls(n = 111)	p-Value
**MEDICAL STUDENTS**			
**Total number of medical notes**	139	111	–
**Number of medical notes with subjective elements**	134	110	–
**Subjective notes/1,000 patient-days**	116.5	114.9	0.90
**RESIDENTS**			
**Total number of medical notes**	452	417	–
**Number of medical notes with subjective elements**	399	399	–
**Subjective notes/1,000 patient-days**	347.0	416.9	0.01
**ATTENDING PHYSICIANS**			
**Total number of medical notes**	653	528	–
**Number of medical notes with subjective elements**	444	432	–
**Subjective notes/1,000 patient-days**	454.5	451.4	0.02
**TOTAL**			
**Total number of medical notes**	1,244	1,056	–
**Number of medical notes with subjective elements**	977	941	–
**Subjective notes/1,000 patient-days**	849.6	983.3	0.001
**Total number of patient-days**	1,150	957	–
**Median daily number of medical notes with subjective element (IQR)**	0.90 (0.69–1.0)	1.0 (0.92–1.3)	0.0001

Also, data not shown in tables showed a tendency towards complications being more preventable (as to their Baker preventability scores) in isolated patients than controls. Eleven (11) complications in the isolated group *vs.* three (3) in the control group had a Baker preventability score greater than 3 (indicator of more preventable complications) (p = 0.06). No difference was obtained regarding causality as to Baker causality scores (score >3, indicator of more preventable complications: 18 vs. 21, p = 0.73). Table 3 shows a description of the complications in each group.

**Table 3:** Complications Description.

Complication Category	Isolated patients (n = 111 patients)	Controls (n = 111 patients)	p-Value
Deaths	10	11	–
Pulmonary-related	2	3	–
Heart-related	6	4	–
Infection-related	2	4	–
Bleeding	5	6	–
Delirium	3	11	–
Immobilisation/falls	7	2	–
Infections	9	3	–
Dysglycemia	0	4	–
Gastro-intestinal	1	3	–
Renal/electrolytes	11	11	–
Arrhythmia	0	5	–
Myocardial infarction	0	2	–
Blood pressure	2	1	–
Heart failure	2	1	–
Medication error	6	0	–
Other	4	2	–
**Total number of complications**	60	62	–
**Mean number of complications per patient, n ± SD**	0.57±1.01	0.59±1.05	0.89
**Complications/1,000 patient-days**	52.2	64.8	0.40

## Discussion

Our results show that cohort isolation is associated with fewer bedside visits from the medical staff than non-isolated ward hospitalisation. Hospital stay could also be longer, and there is a tendency towards complications being more preventable in the isolated group, although we could not show differences in causality or in the total number of complications. This suggests that those adverse events were unrelated to isolation, but may have been prevented by more frequent visits.

Others before us have outlined the negative influence of contact precautions on physical examination by attending physicians on the regular ward and of overall patient contact time in the ICU [9], [10]. Although these series relied on direct observation rather than indirect measures of patient visits, we studied specifically patients hospitalised on a dedicated ward, which had not been done before. We also were able to demonstrate that isolation affects residents’ behavior as well as their attendings’. As mentioned earlier, part of the rationale for cohort isolation is to limit the cumbersome aspects of gowning to promote patient contact. Our results show that this argument falls short, but we are unable to determine if gowning itself is the problem or if other factors reduce the number of visits. Is moving to a different ward to finish their round an important obstacle for physicians?

The therapeutical relationship could also be hampered. Patient contact remains a cornerstone of medical practice and patient care, isolation or not.

Our design allowed us to control for most confounding factors. Our two groups shared similar comorbitity scores, epidemiologic features and admission diagnoses. Hence, it is difficult to elaborate on the trend in the length of stay. It is possible that isolation induced a less favorable perception of the patients (i.e. the isolated patient might have been perceived as “sicker”). This in turn may have influenced the discharge decision, as remission from a COPD exacerbation or heart failure decompensation is mostly a clinical diagnosis.

A growing body of literature outlines the various potential adverse effects of contact precautions. Ethics and psychological side-effects may be an issue [11], [12], not to mention patient safety, as shown in Stelfox’ widely quoted 2004 retrospective study [13]. However, most of these results apply to standard, individual isolation. We were unable to show a statistical difference in our retrospective group of cohorted patients, but overall, we had a small number of events.

The trend in more preventable complications may be explained by the less frequent visits. Although not an adverse event *per se*, not visiting the patient may have lead to the later diagnosis of certain complications (*e.g.* immobilisation and falls, infections). However, because of absence of difference in the total number of complications, our small number of patients and events and the heterogeneity of the results, we decided not to expand our analysis further than reporting the trend.

Our main limitation remains the retrospective nature of our work, confining us to surrogate means of asserting patient visits and to patient charts to analyse potential complications. Hence, we cannot retrospectively determine the number of bedside visits that are not clearly documented in the progress notes. Using subjective notes as a surrogate marker of bedside visits is imperfect, but remains the most valid we could use retrospectively. This method is very specific (patients with subjective notes were most certainly visited), but certainly lacks specificity (all visited patients do not necessarily have a subjective note). However, as discussed earlier, available prospective data in the literature also point towards fewer visits in the isolated population [9], [10].

Of note, we did not compare cohort to standard isolation, but to non-isolated controls. Also, if physicians visit isolated patients’ bedside less frequently, complications may be underreported, therefore introducing a reporting bias in our results.

We did not address the effectiveness of isolation measures or caregivers’ adherence to gowning. We could not explain the reason behind the reduced number of visits either. Many questions remain unanswered, and larger prospective studies are needed to clarify both safety and cost-effectiveness of isolation measures.

### Conclusions

Isolated patients have less documented care that suggests less bedside visits from the medical staff, which could hamper the therapeutical relationship and patient safety. Further studies are needed to explain this finding.
